# Salmonella Utilizes Zinc To Subvert Antimicrobial Host Defense of Macrophages via Modulation of NF-κB Signaling

**DOI:** 10.1128/IAI.00418-17

**Published:** 2017-11-17

**Authors:** Aimin Wu, Piotr Tymoszuk, David Haschka, Simon Heeke, Stefanie Dichtl, Verena Petzer, Markus Seifert, Richard Hilbe, Sieghart Sopper, Heribert Talasz, Dirk Bumann, Cornelia Lass-Flörl, Igor Theurl, Keying Zhang, Guenter Weiss

**Affiliations:** a Department of Internal Medicine II, Medical University of Innsbruck, Innsbruck, Austria; b Animal Nutrition Institute, Key Laboratory for Animal Disease, Sichuan Agricultural University, Ya'an, Sichuan, People's Republic of China; c Department of Internal Medicine V, Medical University of Innsbruck, Innsbruck, Austria; d Biocenter, Division of Clinical Biochemistry, Medical University of Innsbruck, Innsbruck, Austria; e Biozentrum, University of Basel, Basel, Switzerland; f Department of Microbiology and Hygiene, Medical University of Innsbruck, Innsbruck, Austria; University of California, Davis

**Keywords:** NADPH oxidase, NF-κB, Salmonella, macrophages, nitric oxide synthase, zinc

## Abstract

Zinc sequestration by macrophages is considered a crucial host defense strategy against infection by the intracellular bacterium Salmonella enterica serovar Typhimurium. However, the underlying mechanisms remain elusive. In this study, we found that zinc favors pathogen survival within macrophages. Salmonella-hosting macrophages contained higher free zinc levels than did uninfected macrophages and cells that successfully eliminated bacteria, which was paralleled by the impaired production of reactive oxygen species (ROS) and reactive nitrogen species (RNS) in bacterium-harboring cells. A profound, zinc-mediated inhibition of NF-κB p65 transcriptional activity affecting the expression of the ROS- and RNS-forming enzymes phos47 and inducible nitric oxide synthase (iNOS) provided a mechanistic explanation for this phenomenon. Macrophages responded to infection by enhancing the expression of zinc-scavenging metallothioneins 1 and 2, whose genetic deletion caused increased free zinc levels, reduced ROS and RNS production, and increased the survival of Salmonella. Our data suggest that Salmonella invasion of macrophages results in a bacterium-driven increase in the intracellular zinc level, which weakens antimicrobial defense and the ability of macrophages to eradicate the pathogen. Thus, limitation of cytoplasmic zinc levels may help to control infection by intracellular bacteria.

## INTRODUCTION

Macrophages, which constitute the first line of antimicrobial defense, are equipped with a broad repertoire of mechanisms to clear intracellular bacteria (1, 2). One such strategy is to limit the availability of nutrients important for microbial growth, such as iron, copper, manganese, or zinc (3–6). Whereas the role of iron in the host-pathogen interaction in infection by intracellular bacteria such as Salmonella enterica serovar Typhimurium or mycobacteria has been extensively studied (7–9), far less is known about the role of zinc in this setting.

The transition metal zinc (Zn) is essential for a plethora of structural proteins and enzymes and impacts immune cell function and differentiation (10, 11). Zinc deficiency has been linked to impaired B, T, and NK cell responses and inflammatory cytokine production (11). Recent evidence suggests that the accumulation of Zn in the Golgi apparatus of activated macrophages triggers the formation of toxic radicals by NADPH oxidase (NOX), thereby contributing to the clearance of Histoplasma capsulatum (12). An excessive cytokine-induced accumulation of Zn and Cu in bacterium-containing phagosomes can intoxicate intracellular pathogens such as mycobacteria (1, 13). Of note, microbes developed measures to circumvent zinc toxicity (4, 14). Hence, both the compartment-specific sequestration of vital metal ions as well as bacterial intoxication by high metal ion concentrations belong to the defense arsenal of the macrophage (4, 15).

On the other hand, most pathogens require zinc for their metabolic needs and for the defense against host-mediated oxidative stress (16). This may be a reason why neutrophils secret calprotectin, which scavenges zinc and reduces its availability for microbes in the extracellular compartment (4). More evidence for the ambiguous role of zinc in infections comes from randomized clinical trials aiming at improving children's health by correcting Zn and iron deficiencies. In this setting, dietary zinc and iron supplementation correlated with increased morbidity and mortality from infections (17), part of which may be related to Zn-mediated alterations of the intestinal microbiota (18). However, previous studies also indicated a beneficial effect of Zn supplementation on the incidence and outcome of bacterial infections (11, 19).

Salmonella Typhimurium is a Gram-negative bacterium that resides and replicates within macrophages (20, 21). Several studies have shown that its growth and pathogenicity depend strictly on a sufficient supply of iron (22) and that host mechanisms that restrict iron availability can efficiently control Salmonella proliferation in the cell (23, 24). However, much less is known about the importance of macrophage zinc homeostasis in the control of Salmonella infection. In response to pathogen invasion, macrophages induce numerous antimicrobial pathways, including the formation of reactive oxygen species (ROS) through the NAPDH oxidase complex and reactive nitrogen species (RNS) by inducible NO synthase (iNOS) (2, 25–28). Salmonella can dismantle the radical-producing machinery, for example, by reprogramming host metabolism and gene expression via a type III expression system (T3SS) encoded by Salmonella pathogenicity island 2 (SPI-2) (29, 30). The activation of radical-detoxifying enzymes such as Cu/Zn superoxide dismutase provides another measure to resist oxidative and nitrosative stress, for which zinc is actively acquired from the environment by invading pathogens (31, 32).

Here, we shed light on a previously unrecognized role of zinc in the host-pathogen interplay. We found that Salmonella induces the accumulation of protein-unbound zinc (labile/free zinc) in infected macrophages to impair fully fledged NF-κB activation, which is essential for the transcriptional induction of NADPH oxidase and iNOS. This results in dampened ROS and RNS formation leading to improved pathogen survival. We further demonstrate that free zinc mobilization by genetic deletions of zinc-chelating proteins hinders efficacious antibacterial immune defense.

## RESULTS

### Salmonella infection induces free zinc accumulation in infected macrophages.

We utilized the RAW264.7 macrophage cell line and the intracellular bacterium Salmonella Typhimurium to study the impact of cellular zinc availability on host-pathogen interactions.

**FIG 1:**
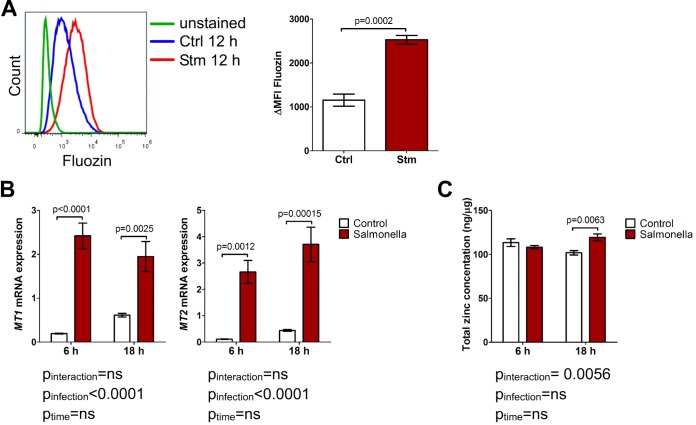
Salmonella infection induces free zinc accumulation in macrophages. RAW264.7 cells were infected with S. Typhimurium (Stm) for the indicated times. (A) Staining with the Fluozin reporter for free zinc. The ΔMFI of the signal was measured by flow cytometry. Representative flow cytometry data with a summary graph (*n* = 4) are presented. (B) *Mt1* and *Mt2* expression was assessed by reverse transcription-quantitative PCR (qRT-PCR) (*n* = 4). (C) The total zinc concentration was measured by atomic absorption spectrometry and normalized to protein levels (*n* = 4). Statistical significance was determined with an unpaired *t* test (A) and two-way ANOVA with a Bonferroni *post hoc* test (B and C). ns, not significant.

Infection of RAW264.7 macrophages with S. Typhimurium resulted in a significant increase in the level of labile intracellular zinc, as measured by Fluozin fluorescence (Fig. 1A). In parallel, the mRNA expression of two important Zn-binding metallothioneins (MTs), MT1 and MT2 (33), was induced, which could be considered a host response mechanism to limit labile zinc levels in cells (Fig. 1B). Of interest, we detected only a minor increase in the total (i.e., free and protein-bound) cellular zinc content following infection using atomic absorption spectrometry (Fig. 1C). This suggests that primarily a shift of zinc from protein-bound sources to the free intracellular zinc pool, rather than the acquisition of the metal from the extracellular space, may occur in macrophages upon Salmonella infection.

### Zinc supplementation promotes Salmonella infection in macrophages by impairing clearance of the pathogen.

**FIG 2:**
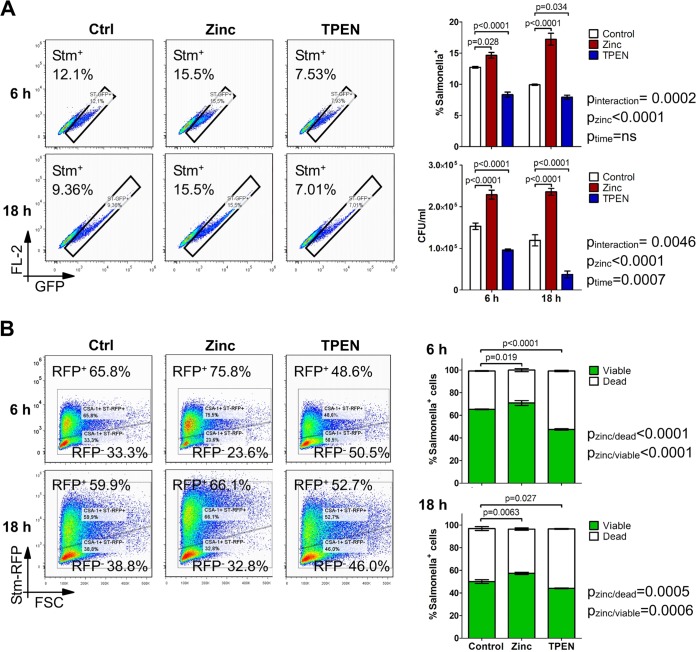
Zinc supplementation increases the infection rate by impairing the killing capacities of macrophages. RAW264.7 cells were stimulated with the vehicle, zinc, or TPEN and infected with GFP-expressing (A) and RFP-expressing (B) S. Typhimurium strains for the indicated time points. (A) The percentage of Salmonella-containing cells among viable macrophages (DAPI^−^) was determined by flow cytometry and confirmed by plating of lysed cell cultures. Representative cytometry plots of DAPI^−^ macrophages are presented with a summary graph (*n* = 4). (B) Cells were stained intracellularly with an anti-CSA antibody recognizing both live and killed Salmonella. The percentages of macrophages containing RFP^+^ live and RFP^−^ dead bacteria among CSA^+^ cells were determined by flow cytometry. Representative data and a summary graph (*n* = 3) are shown. Statistical significance was assessed by two-way (A) and one-way (B) ANOVAs with Bonferroni *post hoc* tests. FSC, forward scatter.

To study the biological importance of altered zinc availability for the course of Salmonella infection in macrophages, we modulated zinc availability by adding either ZnCl_2_ or *N*,*N*,*N*′,*N*′-tetrakis(2-pyridylmethyl)ethylenediamine (TPEN) (34), which specifically chelates intracellular zinc, to macrophages infected with green fluorescent protein (GFP)-expressing Salmonella bacteria (see Fig. S1A in the supplemental material). Neither of the treatments diminished cell viability (Fig. S1B). Interestingly, zinc supplementation significantly elevated the percentage of Salmonella-infected macrophages and total numbers of bacteria (Fig. 2A and Fig. S2A). In turn, zinc chelation had an opposite effect on both readouts of infection (Fig. 2A). Of note, the same effects of zinc modulation on the infection rate were observed in mouse primary bone marrow-derived macrophages (Fig. S3). This observation could not be explained by an alteration of the phagocytic uptake of particles such as bacteria or beads, as zinc supplementation decreased rather than increased the phagocytic capacity of macrophages (Fig. S2B and S2C). This led us to hypothesize that the infection-promoting action of increased cellular zinc levels occurs after the entry of the bacteria into the cell. Zinc addition had no effects on bacterial survival and proliferation in *in vitro* cultures, hence excluding the possibility that additional zinc favors extra- or intracellular bacterial growth, resulting in a higher macrophage infection rate (Fig. S4).

To test whether free zinc directly alters intracellular bacterial proliferation, we determined the number of viable bacteria per cell following ZnCl_2_ or TPEN treatment. Relative numbers of bacteria per cell remained unaffected upon this treatment (Fig. S5A), suggesting that not intracellular pathogen proliferation but rather the susceptibility of macrophages to bacterial invasion is increased upon higher zinc availability.

We then sought to test whether zinc can inhibit the immediate killing of Salmonella by macrophages. We thus quantified the percentages of macrophages containing viable and dead bacteria upon zinc modulation and infection with the red fluorescent protein (RFP)-expressing Salmonella reporter strain. Intracellular bacteria were additionally stained with an anti-CSA-1 antibody, which recognizes both live and dead Salmonella bacteria. Since only metabolically active bacteria produce RFP, we defined viable Salmonella bacteria as being CSA-1 positive (CSA-1^+^) RFP^+^ and dead ones as being CSA-1^+^ RFP negative (RFP^−^) (Fig. S5B). As shown in Fig. 2B, surplus zinc significantly reduced the number of macrophages containing dead bacteria, whereas TPEN treatment increased it. These data led us to hypothesize that an increase in the level of free zinc in macrophages may be employed by Salmonella to resist the antimicrobial elimination pathways of the host.

### Zinc supplementation affects Salmonella clearance through inhibition of ROS and RNS production.

The generation of ROS and RNS by NOX and iNOS in macrophages serves as an efficacious mechanism to eliminate intracellular pathogens (2, 22). These pathways are also of importance in our *in vitro* infection system, since treatment of macrophages with a ROS scavenger, *N*-acetyl-cysteine (NAC), or an iNOS inhibitor, *N*^6^-(1-iminoethyl)-l-lysine (l-NIL), increased the numbers of intracellular bacteria (details not shown).

**FIG 3:**
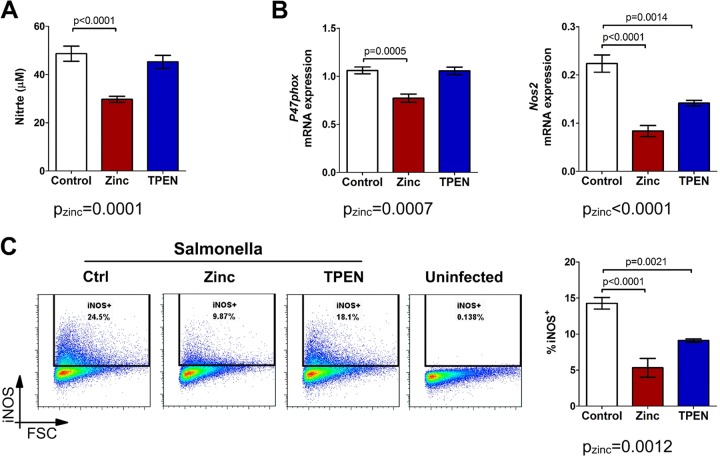
Free cellular zinc inhibits ROS and RNS production during Salmonella infection. RAW264.7 cells were stimulated with the vehicle, zinc, or TPEN and infected with S. Typhimurium for 6 h (A and B) or 18 h (C). (A) Nitrite concentrations in cell culture supernatants were determined with the Griess assay (*n* = 8). (B) Expression of the *p47phox* and *Nos2* genes was assessed by qRT-PCR (*n* = 4). (C) Infected macrophages were stained for intracellular iNOS, and percentages of iNOS^+^ cells were determined by flow cytometry. Representative flow cytometry plots are presented together with a summary graph (*n* = 3). Statistical significance was assessed by two-way ANOVA with a Bonferroni *post hoc* test.

Since elevated free zinc levels hindered the effective clearance of Salmonella, we investigated whether the modulation of zinc concentrations affected ROS and RNS production upon bacterial infection. We detected a drop in nitrite production (the stable end product of the NO pathway) after zinc supplementation (Fig. 3A). The formation of ROS and RNS is tightly controlled by the transcriptional, posttranscriptional, and posttranslational regulation of ROS/RNS-generating enzymes. Upon zinc supplementation, the mRNA levels of iNOS and the key NOX subunit p47phox were significantly reduced (Fig. 3B). Accordingly, a significant reduction of the iNOS protein level was observed as well (Fig. 3C). Of note, treatment with TPEN did not increase p47phox mRNA and iNOS transcript and protein amounts above the control levels, suggesting that the TPEN-mediated improvement of the elimination of bacteria may also involve some other radical-independent antimicrobial pathways.

To test for the possibility that zinc stimulation can protect bacteria against the detrimental reactive oxygen and nitrogen species in a macrophage-independent manner, we monitored the growth of wild-type (WT) Salmonella bacteria in the presence of ROS (paraquat) and RNS (*S*-nitroso-*N*-acetyl-dl-penicillamine [SNAP]) generators with zinc and TPEN supplementation. As shown in Fig. S6 in the supplemental material, neither the addition of zinc nor its chelation impacted the survival and proliferation of the bacteria under conditions of oxidative and nitrosative stress.

### Zinc inhibits NF-κB activation.

**FIG 4:**
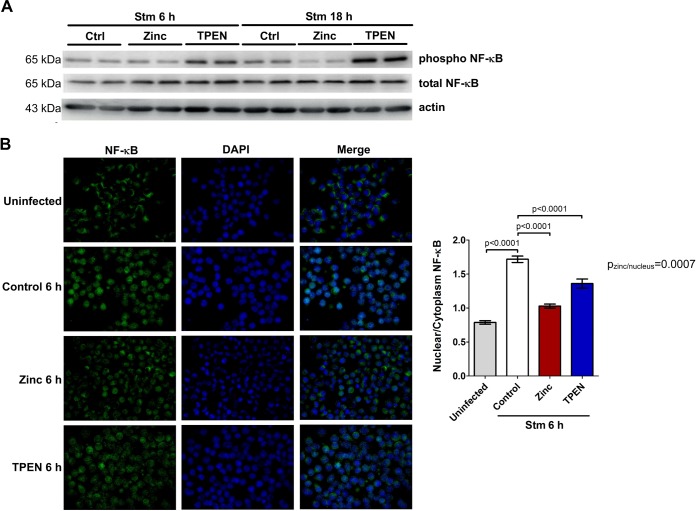
Increases in free zinc concentrations hamper NF-κB signaling. RAW264.7 cells were stimulated with the vehicle, zinc, or TPEN and infected with S. Typhimurium for the indicated times. (A) Levels of pSer536 NF-κB and total NF-κB in whole-cell lysates were measured by Western blotting. Actin served as a loading control. Representative results of one experiment are shown (*n* = 2). (B) The immunofluorescent NF-κB signal intensity in the nucleus and cytoplasm was measured by fluorescence microscopy. Representative images originating from one representative experiment are shown (*n* = 2). Data presented in the graph refer to signal intensities in at least 10 high-power fields under each experimental condition. Statistical significance was calculated by one-way ANOVA with a Bonferroni *post hoc* test.

Nuclear factor kappa B (NF-κB) is a master regulator of iNOS and p47phox mRNA expression (2, 35). Having observed that surplus zinc downregulates both of these transcripts, we asked whether zinc can affect NF-κB transcriptional activity. The phosphorylation and nuclear accumulation of the p65 subunit are surrogates for NF-κB activation. The addition of zinc reduced p65 phosphorylation in infected macrophages, whereas zinc chelation strongly enhanced it (Fig. 4A). Similarly, we observed impaired p65 nuclear *trans*-localization after zinc addition and the opposite phenomenon after TPEN treatment (Fig. 4B; see also Fig. S7 in the supplemental material). To further corroborate the impact of zinc on the transcriptional activity of p65 NF-κB, we performed a chromatin immunoprecipitation assay with a phospho-p65 antibody. As shown in Fig. S5A in the supplemental material, zinc almost completely abrogated p65 binding to the proximal site in the *iNos* promoter after Salmonella infection as well as to promoters of other classical p65 target genes, *Icam* and *Cxcl2*. Zinc chelation in turn increased the affinity of p65 for both binding sites in the *iNos* promoter region (Fig. S8A). Our finding that zinc supplementation impairs p65 transcriptional activity in general is further supported by the effects of surplus zinc on mRNA levels of the NF-κB-regulated cytokine genes *Il1b*, *Il6*, *Il10*, and *Tnf* (Fig. S8B). Notably, these effects were specific for the NF-κB pathway, since the activity of extracellular signal-regulated kinase (ERK), STAT1, and STAT3 signaling remained unchanged (Fig. S9).

Taken together, these data show that the expansion of cellular zinc levels impairs the killing capacity of macrophages by inhibiting NF-κB activation and the downstream transcriptional activation of iNOS, phox47, and several proinflammatory cytokines.

### Salmonella uses free zinc to overcome host clearance.

As an intracellular pathogen, Salmonella developed a plethora of strategies to bypass antimicrobial effector pathways of macrophages. We hypothesized that the cellular accumulation of free cytoplasmic zinc causing the inhibition of ROS/RNS formation may pose such a bacterium-driven protective mechanism.

**FIG 5:**
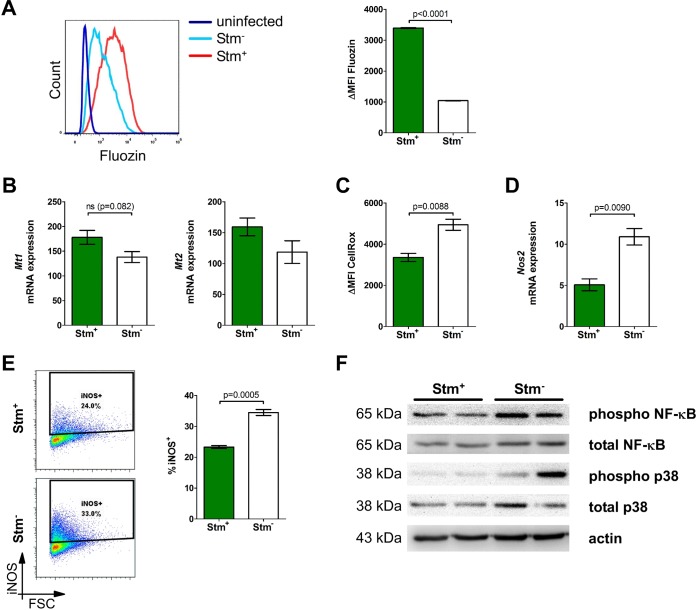
Zinc accumulation can pose a pathogen's strategy to evade clearance in macrophages. RAW264.7 cells were infected with RFP-expressing S. Typhimurium for 6 h. Viable cells (DAPI^−^) containing live Salmonella (Stm^+^) and no/dead Salmonella (Stm^−^) bacteria were analyzed by flow cytometry and sorted by using a fluorescence-activated cell sorter. (A) Free zinc levels were determined by Fluozin staining. Representative Fluozin signal histograms are shown. The plot depicts the ΔMFI (*n* = 3). (B) *Mt1* and *Mt2* gene expression levels measured by qRT-PCR in fluorescence-activated cell sorter-sorted DAPI^−^ *S*. Typhimurium-positive and DAPI^−^ *S*. Typhimurium-negative macrophages (*n* = 3). (C) ROS production was measured by CellROX staining. The plot depicts the ΔMFI (*n* = 3). (D) *Nos2* gene expression was measured by qRT-PCR in fluorescence-activated cell sorter-sorted viable *S*. Typhimurium-positive and *S*. Typhimurium-negative macrophages (*n* = 3). (E) Percentages of cells positive for the intracellular iNOS protein were determined by flow cytometry. Representative cytometry plots are shown with a summary graph (*n* = 3). (F) pSer536 NF-κB, total NF-κB, phospho-p38, and total p38 levels were measured in fluorescence-activated cell sorter-sorted DAPI^−^ *S*. Typhimurium-positive and DAPI^−^ *S*. Typhimurium-negative macrophages by Western blotting. Results of a representative experiment are presented (*n* = 2). Statistical significance was assessed by two-way ANOVA with a Bonferroni *post hoc* test.

To test this, we infected macrophages with RFP reporter-expressing Salmonella bacteria and sorted bacterium-hosting (RFP^+^) and bacterium-cleared/uninfected (RFP^−^) macrophages from these cultures. Macrophages containing viable Salmonella bacteria accumulated significantly more free zinc (Fig. 5A) and expressed more zinc-sensitive *Mt1* and *Mt2* transcripts (Fig. 5B) than did Salmonella-naive or Salmonella-clearing macrophages. As with zinc supplementation, we also observed significantly reduced ROS production (Fig. 5C) and iNOS mRNA (Fig. 5D) and iNOS protein (Fig. 5E) expression levels in Salmonella-hosting macrophages compared to bacterium-negative cells. Importantly, this was paralleled by a profound downregulation of phosphorylated p65 and an inhibition of the downstream p38 pathway in RFP^+^ macrophages compared to Salmonella-clearing ones (Fig. 5F). No such consistent regulatory pattern was seen among other signaling pathways involved in antibacterial activity, such as STAT1, STAT3, and ERK (see Fig. S10 in the supplemental material). These data support the hypothesis that either increased zinc levels in macrophage are a predisposing factor for Salmonella infection or Salmonella invasion of macrophages may promote cytoplasmic zinc accumulation, which impairs the immediate NF-κB-mediated antimicrobial host defense.

### Mt1/2 knockout in macrophages favors intracellular Salmonella survival.

**FIG 6:**
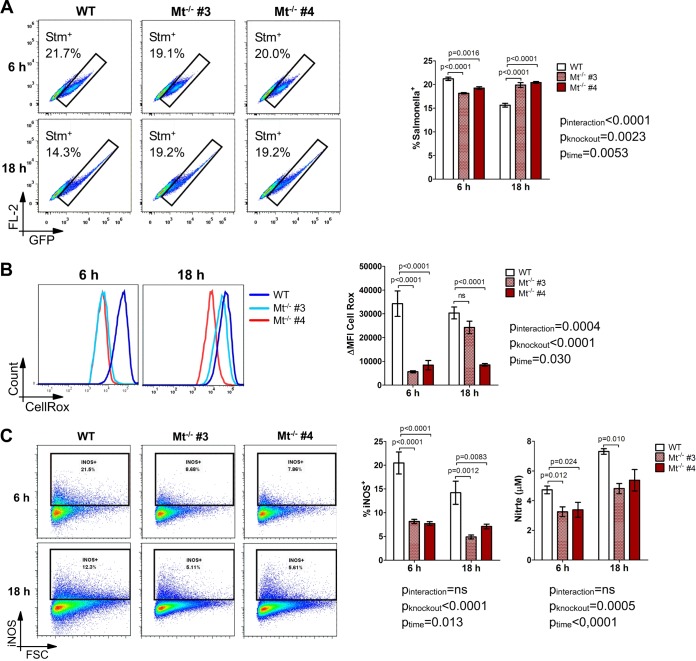
Genetic elevation of free zinc levels by *Mt* gene knockout inhibits ROS and RNS production and bacterial clearance. Double-knockout Mt1/2^−/−^ cells and parental WT RAW264.7 cells were infected with GFP-expressing S. Typhimurium for the indicated times. (A) The percentage of Salmonella-containing cells among viable macrophages (DAPI^−^) was determined by flow cytometry. Representative cytometry plots of DAPI^−^ macrophages are shown with a summary graph (*n* = 3). (B) Levels of cellular ROS among viable macrophages were measured by CellROX staining and flow cytometry (DAPI^−^). Representative signal histograms are shown. The plot depicts ΔMFIs (*n* = 3). (C) Percentages of cells positive for intracellular iNOS protein were measured by flow cytometry. Representative cytometry plots and a summary graph are shown (*n* = 3). The nitrite concentration in the cell culture supernatant was determined with the Griess assay (*n* = 4). Statistical significance was ascertained by two-way ANOVA with a Bonferroni *post hoc* test.

The zinc-binding metallothioneins MT1 and MT2 are vital regulators of cellular free zinc levels. Their expression is induced upon Salmonella infection and specifically increased in Salmonella-hosting macrophages. We hypothesized that the upregulation of MT1 and MT2 may pose a host response mechanism to limit zinc availability for intracellular bacteria. To study their specific role in our infection model, we disrupted the *Mt1* and *Mt2* genes in macrophages using the clustered regularly interspaced short palindromic repeat (CRISPR)-Cas9 technique (see Fig. S11A in the supplemental material). This event results in a boost of cellular free zinc levels, even without zinc supplementation, in resting and infected cells (Fig. S11B and S11C). Comparably to zinc supplementation, *Mt* knockout cells displayed increased Salmonella colonization (Fig. 6A and Fig. S11D), reduced ROS levels (Fig. 6B), and impaired RNS production as assessed by nitrite and iNOS protein levels (Fig. 6C) compared with wild-type macrophages.

These results further corroborate the key impact of labile intracellular zinc on intracellular bacterial survival by modulating antimicrobial ROS and RNS formation in Salmonella-infected macrophages. In addition, we identify infection-driven MT1 and MT2 expression with the subsequent reduction of intracellular zinc levels as an important antibacterial host response mechanism.

## DISCUSSION

The influence of infection-induced alterations of zinc homeostasis on the course of inflammatory processes and the efficacy of bacterial clearance is largely unknown. In this study, we examined the role of zinc availability in the control of infection by the intracellular bacterium Salmonella Typhimurium and its impact on macrophage-mediated antimicrobial effector pathways. Both the excessive accumulation of zinc in pathogen-containing cell compartments as well as its restriction from invading pathogens were previously described to constrain the growth of various intra- and extracellular microbes (1, 4, 11–14, 16–19).

Here, we observed that Salmonella-infected macrophages display higher free zinc levels than do cells unexposed to the pathogen or macrophages that successfully eliminated bacteria. However, the rise in the amount of total cellular zinc was marginal, suggesting a shift from protein-bound zinc to the free-ion compartment rather than the active uptake of the metal into cells. To our surprise, zinc supplementation promoted neither bacterial intoxication nor clearance but instead increased the vulnerability of RAW264.7 as well as murine primary bone marrow-derived macrophages to infection. This was directly linked to the reduced formation of antibacterial ROS and RNS in zinc-supplemented macrophages (2, 36, 37). Free zinc inhibited p65 NF-κB activation, hence dampening the transcriptional expression of the ROS- and RNS-generating enzymes p47phox and iNOS (38, 39). This is in agreement with the previously described inhibitory effect of zinc on other NF-κB-driven effector pathways (40–43), which impacted the immune response in polymicrobial sepsis (44). Therefore, zinc was proposed to upregulate the NF-κB inhibitor A20 and to bind directly to IκB kinase beta (IKKB), hence moderating its activity (45, 46). We further showed that Zn diminishes p38 and p65 phosphorylation and, consequently, NF-κB transcriptional activity, hence blunting antimicrobial host responses.

Also, Zn may act as a cofactor of antioxidative enzymes such as Zn/Cu superoxide dismutase (SOD) (31, 32), which also protect microbes from oxidative damage by host-generated radicals. However, this possibility seems unlikely, since zinc supplementation did not modify bacterial growth in the presence of ROS and RNS generators in a macrophage-free setting.

We thus propose that the accumulation of free zinc in macrophages serves as a protective mechanism enabling bacteria to withstand killing by macrophages (10, 26). Using a fluorescent reporter-expressing Salmonella strain, we found that macrophages hosting viable bacteria accumulate dramatically higher free zinc levels than do cells that cleared pathogens, which was paralleled by the downregulation of ROS and RNS formation. However, the underlying mechanism for cellular Zn mobilization remains elusive. Nevertheless, Salmonella may use the increased free zinc levels for its own purposes, such as dismantling the killing machinery. Our data obtained with the *Mt1/2* knockout cell lines not only underline the crucial role of free zinc in infected cells but also stress the vital contribution of metallothioneins to the inhibition of bacterial colonization (47). The robust induction of MT1 and MT2 by zinc supplementation, inflammatory stimuli, or bacterial products may be seen as a defense strategy of macrophages to reduce the concentration of free zinc ions in the cell and hence enable the fully fledged NF-κB-mediated cytokine response and bacterial clearance (41, 48). However, there were only nonsignificant differences in the expression levels of MT1 and MT2 transcripts within macrophages containing either viable or dead bacteria, which points to the need to not only evaluate MT expression dynamically over time but also study the functional activity of MT proteins in these settings.

In summary, we propose a novel role for labile cytoplasmic zinc in the course of infection by intracellular bacteria such as Salmonella. Zinc accumulation in macrophages blunts NF-κB activation and impairs NF-κB-dependent bacterial clearance mediated by iNOS and p47phox. This phenomenon can also be regarded as a strategy of Salmonella and possibly other intracellular bacteria to overcome oxidative and nitrosative stress in macrophages. Therapeutic strategies that reduce zinc accumulation in cells or that increase the zinc-scavenging capacity of metallothioneins may prove effective for the treatment of infections by intracellular microbes.

## MATERIALS AND METHODS

### Bacterial strains.

Wild-type Salmonella enterica serovar Typhimurium bacteria were obtained from the ATCC (ATCC 14028). Reporter GFP-expressing (ST-GFP) and RFP-expressing (ST-RFP) Salmonella strains were described previously (49, 50).

### Cell culture and Salmonella infection *in vitro*.

The murine macrophage cell line RAW264.7 was kept in Dulbecco's modified Eagle's medium (DMEM) with 2 mM l-glutamine and 10% fetal calf serum (FCS) (Biochrom). Cells were routinely collected by scraping. Cells were seeded at a density of 5 × 10^4^ cells/cm^2^ a day before infection. Infections with WT, ST-GFP, and ST-RFP Salmonella bacteria were performed at a multiplicity of infection (MOI) of 1:10, as described previously (23), 1 h after bacterial challenge medium was changed to a gentamicin-containing one (25 μg/ml). A total of 100 μM ZnCl_2_ or 5 μM TPEN (Sigma) was added to the cultures concomitantly with the bacteria and was present for the whole infection period.

### Flow cytometry, cell sorting, and fluorescence microscopy.

Flow cytometry measurements were performed with a Beckman-Coulter Gallios device and analyzed with FlowJo software (FlowJo LLC). ΔMFI is defined as the difference in the signal intensity between an unstained control and a stained sample. For sorting, a FACSAria I device (Becton Dickinson) was used. Microscopy images were acquired with an Axioskop fluorescence microscope (Zeiss) and analyzed with ImageJ software.

### Determination of free and total zinc.

Macrophage cultures were incubated with 5 μM Fluozin 3-AM (Life Technologies) for 1 h at 37°C. Subsequently, medium was removed, and cells were incubated in phosphate-buffered saline (PBS) for 30 min at 37°C. The green fluorescence of the probe was detected by fluorescence microscopy and flow cytometry. Total zinc concentrations were measured by atomic absorption spectrometry as described previously (51).

### Quantitative real-time PCR and Western blotting.

RNA was isolated and reverse transcribed as described previously (52). Relative gene expression was calculated with the ΔΔ*C_T_* method, normalizing the results to the value for the *Hprt* gene. The list of primer oligonucleotide sequences can be found in Table S1 in the supplemental material. Protein extraction and Western blotting were performed as described previously (52), using primary rabbit antibodies against NF-κB p65 (catalog no. 4764), pSer536–NF-κB p65 (catalog no. 3033), p38 (catalog no. 9212), phosphorylated p38 (p-p38) (catalog no. 4511), ERK (catalog no. 4695), p-ERK (catalog no. 4370), STAT1 (catalog no. 9172), p-STAT1 (catalog no. 9167), STAT3 (catalog no. 9132), p-STAT3 (catalog no. 9134), and actin (catalog no. A2066) (all from Cell Signaling Technology, except for actin, which was purchased from Sigma-Aldrich). The dilution of primary antibodies was 1:1,000. As a secondary antibody, a goat anti-rabbit antibody conjugated to horseradish peroxidase (HRP) (catalog number P0448; Dako) was used (1:2,000).

### Immunofluorescent staining of NF-κB.

Salmonella-infected cells were fixed with 4% formalin in PBS for 15 min at room temperature, washed twice with PBS, and blocked in blocking buffer (5% horse serum and 0.3% Triton X-100 in PBS) for 1 h. Cells were stained with a 1:200 dilution of a primary antibody against NF-κB p65 (catalog no. 4764; Cell Signaling Technologies), diluted in antibody dilution buffer (1% bovine serum albumin [BSA] and 0.3% Triton X-100 in PBS) at 4°C overnight. Next, staining with a 1:200 dilution of Dylight488 donkey anti-rabbit IgG (BioLegend) for 1 h was performed, followed by 3 PBS wash steps and mounting with 4′,6-diamidino-2-phenylindole (DAPI)-containing fluorescence mounting medium (Dako). Cytoplasmic and nuclear NF-κB mean fluorescence intensities were measured by using ImageJ software (see Fig. S4 in the supplemental material).

### Intracellular flow cytometry staining and Salmonella killing assay.

Macrophages infected with RFP-expressing Salmonella bacteria were harvested and fixed with 4% formalin in PBS for 15 min at room temperature. Fixed cells were permeabilized with permeabilization buffer (0.05% Triton X-100 in PBS) at room temperature for 15 min. For intracellular iNOS staining, cells were incubated for 1 h with a 1:200 dilution of allophycocyanin (APC)–anti-iNOS antibody (eBioscience). For the killing assay, cells were incubated for 2 h with a 1:200 dilution of biotinylated antibody to Salmonella CSA-1 (catalog no. 16-91-99; KPL) in permeabilization buffer, followed by secondary staining with a 1:200 dilution of Alexa Fluor 647-streptavidin (BioLegend) for an additional 2 h.

### Quantification of reactive oxygen species and nitrite formation.

For the detection of ROS, 5 μM CellROX Deep Red reagent (Life Technologies) was incubated with macrophage cultures for 30 min at 37°C. Subsequently, cells were washed three times with PBS and analyzed by flow cytometry. The nitrite concentration was determined with the Griess assay (Sigma) according to the manufacturer's instructions.

### CRISPR-Cas9 knockout of MT-1.

The metallothionein genes *Mt1* (Entrez identification no. 17748) and *Mt2* (Entrez identification no.17750) were disrupted in RAW264.7 macrophages by using the CRISPR-Cas9 technique (53, 54). In brief, two guide RNAs (gRNAs) targeting the first exon of the *Mt1* and *Mt2* genes, TAGTCGTTGGACGAGTCC (Mt#3) and TCCGAGATCTGGTGAAGC (Mt#4), along with the appropriate restrictase adapters and complementary oligonucleotides were designed. The annealed gRNA duplexes were cloned into the lentiCRISPRv2 vector (a gift from Feng Zhang; Addgene plasmid 52961) (55) by using the BsmBI restriction site. The gRNA-expressing vectors were transfected into HEK293T cells, and cell culture supernatants containing viral particles were harvested and used for infection of RAW264.7 cells. RAW264.7 cells were subsequently subjected to puromycin selection for 3 weeks. Selected clones were tested for *Mt1* and *Mt2* mRNA expression by reverse transcription-PCR (RT-PCR). A representative clone from each of the two CRISPR constructs was used in the experiments described here.

### Statistical analysis.

Each of the experiments described here was performed in at least two independent biological replicates. A replicate utilizing macrophages and bacteria of different passages or batches is defined as a biological replicate. *n* in the figure legends refers to the number of biological replicates.

Statistical analysis was performed by using GraphPad Prism and R software. All results are presented as means ± standard errors of the means (SEM). Statistical tests included unpaired 2-tailed Student's *t* test and one-way or two-way analysis of variance (ANOVA), as appropriate, with Bonferroni *post hoc* tests. A Kolmogorov-Smirnov test was used to compare distributions. *P* values of <0.05 were considered to be statistically significant. On the graphs, *P* values of the main ANOVA effects as well as significant *P* values from *post hoc* tests are presented.

## Supplementary Material

Supplemental material
