# Supplementary material for: Salmonella Utilizes Zinc To Subvert Antimicrobial Host Defense of Macrophages via Modulation of NF-κB Signaling
Source: Infect Immun. 2017 Nov 17;85(12):e00418-17. doi: 10.1128/IAI.00418-17 (PMC5695101; doi:10.1128/IAI.00418-17)
Supplement: Supplemental material [file supp_85_12_e00418-17__index.html]

Supplemental material 

# Salmonella Utilizes Zinc To Subvert Antimicrobial Host Defense of Macrophages via Modulation of NF-κB Signaling

## Supplemental material

- Supplemental file 1 -

  Supplemental methods. Table S1. List of oligonucleotides used for qRT-PCR in this study. Fig. S1. Measurement of free cellular zinc and cell viability under zinc supplementation and depletion. Fig. S2. Identification of *Salmonella*-hosting macrophages by flow cytometry. Phagocytosis capabilities of macrophages upon free zinc modulation. Fig. S3. Effects of cellular zinc level modulation on infection rate and antimicrobial defense in BMDMs. Fig. S4. Effects of zinc addition and chelation on bacterial growth *in vitro*. Fig. S5. Intracellular proliferation of *Salmonella* and strategy for identification of cells hosting viable bacteria and cells clearing the pathogen with flow cytometry. Fig. S6. Influence of zinc modulation on resistance of *Salmonella* Typhimurium to oxidative and nitrosative stress. Fig. S7. Analysis of fluorescence microscopy data to determine nuclear and cytoplasmatic NF-κB signal intensities. Fig. S8. NF-κB transcriptional activity and expression of NF-κB target genes. Fig. S9. Activation of STAT and ERK signaling pathways in *Salmonella*-infected macrophages upon zinc modulation. Fig. S10. Activation of STAT and ERK signaling pathways in *Salmonella*-hosting and *Salmonella*-clearing/noninfected macrophages. Fig. S11. Free zinc accumulation in Mt1/2−/− macrophages. Infection rate in WT and Mt1/2−/− macrophages measured by plating.

  PDF, 6.0M
