# Supplementary material for: Salmonella Utilizes Zinc To Subvert Antimicrobial Host Defense of Macrophages via Modulation of NF-κB Signaling
Source: Infect Immun. 2017 Nov 17;85(12):e00418-17. doi: 10.1128/IAI.00418-17 (PMC5695101; doi:10.1128/IAI.00418-17)
Supplement: Supplemental material [file IAI.00418-17_zii999092204s1.pdf]

Supplemental Material

Supplementary Methods

Chromatin immunoprecipitation (ChIP)

The ChIP was performed essentially as described(1). One milligram chromatin and 1:50 anti-pSer536-NF-κB p65 (#3033, Cell Signaling) or 1:50 control IgG (anti-actin, #4970, Cell Signaling) were used for each IP reaction. Immune complexes were pulled down with 25 µL Protein G Plus Agarose Beads (Santa Cruz Biotech) and DNA isolated with a commercially available kit (High Pure PCR Product Purification Kit, Roche). Input signal and promoter occupancy was determined by Real Time PCR with previously published oligonucleotides (2–4): *iNos* -972 (TCCCAGTTTTGAAGTGACTACG and CATAACTGTTCCCAAAGGGAGA), *iNos* -85 (CACACAGACTAGGAGTGTCCATCAT and CATAACTGTTCCCAAAGGGAGAGT), *Icam* -228 (AGGGGACTAGGCAGTAGTCAATCAG and GAACGAGGGCTTCGGTATTT) and *Cxcl2* -70 (AGGGCAGGGCAGTAGAATGA and TGTGGCTGGAGTCTGGAGTG). Relative binding to the particular promoter site was determined as percent of the ChIP reaction input. In each case the specific signal was at least three times higher as with the control antibody immunoprecipitation.

Bone marrow-derived macrophages

Bone marrow cells were obtained from 2 month old wild-type C57Bl/6N male mice by flushing femurs and tibiae with PBS. Collected cells were subjected to erythrocyte lysis, washed with PBS and cultivated with 50 ng/ml M-CSF (Peprotech) in 10% FCS and PenStrep-containing D-MEM culture medium for 7 days. The medium was renewed every two days.

In vitro Salmonella growth studies

Wild-type *Salmonella* Typhimurium was cultivated overnight to stationary phase (optical density at 600 nm (OD_{600nm})>3.0) in Luria-Bertani (LB) broth. The stationary cultures were diluted to OD_{600nm} in 10 ml LB medium supplemented with indicated concentrations of $ZnCl_2$, TPEN, paraquat (Sigma-Aldrich) or

S-Nitroso-N-acetyl-DL-penicillamine (Sigma) for 8 hours at 37 degree in 50 ml conical tubes with orbital shaking. Every 60 minutes OD_{600nm} values were measured (D30 BioPhotometer, Eppendorf).

Supplementary References

1. **Tamassia N**, **Castellucci M**, **Rossato M**, **Gasperini S**, **Bosisio D**, **Giacomelli M**, **Badolato R**, **Cassatella MA**, **Bazzoni F**. 2010. Uncovering an IL-10-dependent NF-kappaB recruitment to the IL-1ra promoter that is impaired in STAT3 functionally defective patients. FASEB J **24**:1365–75.

2. **Guo H**, **Mi Z**, **Kuo PC**. 2008. Characterization of short range DNA looping in endotoxin-mediated transcription of the murine inducible nitric-oxide synthase (iNOS) gene. J Biol Chem **283**:25209–25217.

3. **Moreno R**, **Sobotzik J-M**, **Schultz C**, **Schmitz ML**. 2010. Specification of the NF-kappaB transcriptional response by p65 phosphorylation and TNF-induced nuclear translocation of IKK epsilon. Nucleic Acids Res **38**:6029–6044.

4. **Tsutsuki H**, **Yahiro K**, **Suzuki K**, **Suto A**, **Ogura K**, **Nagasawa S**, **Ihara H**, **Shimizu T**, **Nakajima H**, **Moss J**, **Noda M**. 2012. Subtilase cytotoxin enhances Escherichia coli survival in macrophages by suppression of nitric oxide production through the inhibition of NF-kappaB activation. Infect Immun **80**:3939–3951.

Name	Sequence
MT1-Fd	5'-CGTGCTGTGCCTGATGTG-3'
MT1-Rv	5'-GGAAGACGCTGGGTTGGT-3'
MT2-Fd	5'-TTCAACCGCCGCCTCCACT-3'
MT2-Rv	5'-AGCACTTCGCACAGCCCAC-3'
iNOS-Fd	5'-CAGCTGGGCTGTACAAACCTT-3'
iNOS-Rv	5'-CATTGGAAGTGAAGCGTTTCG-3'
iNOS-Probe	5'-CGG GCA GCC TGT GAG ACC TTT GA-3'
PHOX47-Fd	5'-GAGGCGGAGGATCCGG-3'
PHOX47-Rv	5'-TCTTCAACAGCAGCGTACGC-3'
PHOX47-Probe	5'-CAACTACGCAGGTGAACCGTATGTAACCATCA-3'
HPRT-Fd	5'-GACCGGTCCCGTCATGC-3'
HPRT-Rv	5'-TCATAACCTGGTTCATCATCGC-3'
HPRT-Probe	5'-ACCCGCAGTCCCAGCGTCGTC-3'

Supplementary Table S1. List of oligonucleotides used for qRT PCR reactions in this study

Supplementary Figure S1

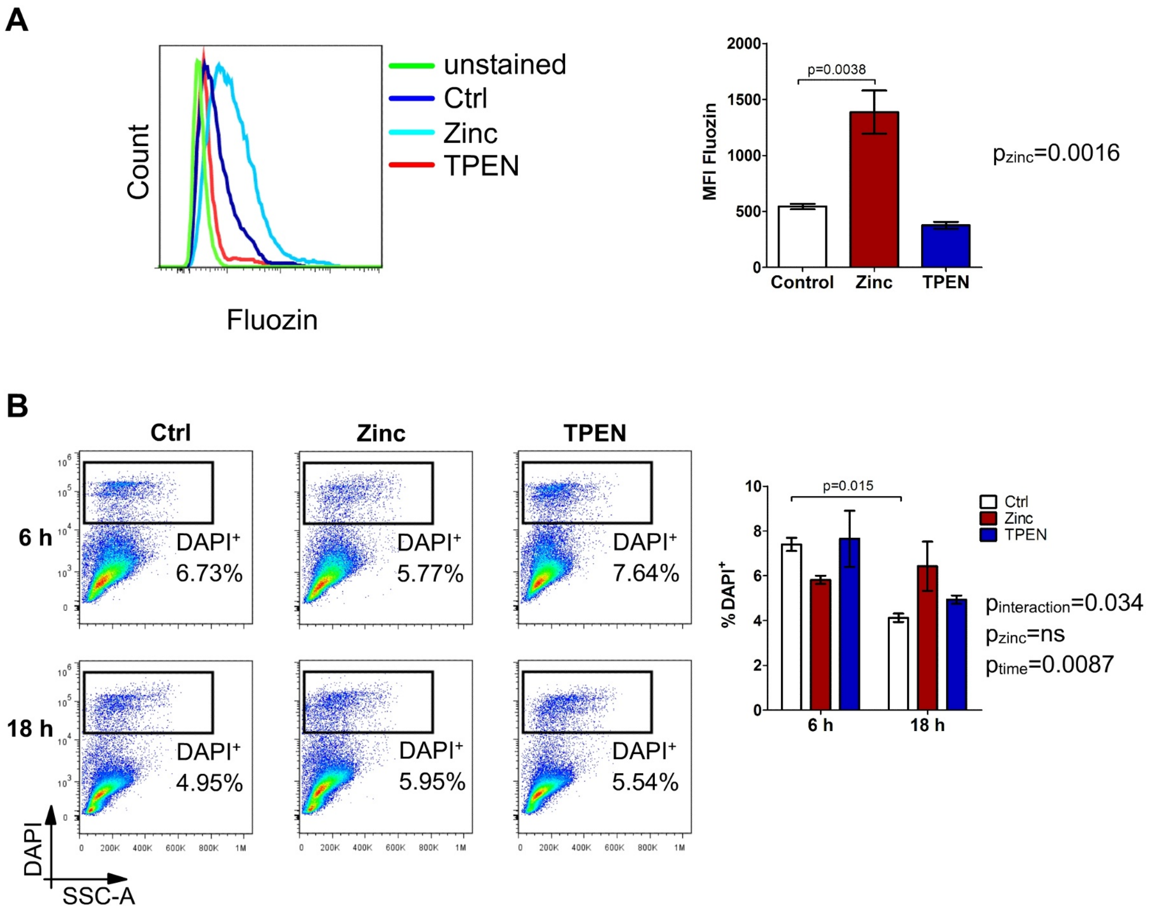

Figure S1. Measurement of free cellular zinc and cell viability under zinc supplementation and depletion. (A) RAW264.7 cells were stimulated with vehicle, zinc or TPEN for 6 h. Accumulation of free zinc in the cell was measured by Fluozin staining and flow cytometry. Representative histograms of Fluozin signal are shown. The graph presents ΔMFI values (n=3). (B) RAW264.7 cells were infected with *S.* Typhimurium and concomitantly stimulated with vehicle, zinc or TPEN for the indicated time points. Cell viability was assessed with DAPI staining and flow cytometry. Representative flow cytometry results are shown with a summary graph (n=4). Statistical significance was calculated with one-way (A) and two-way ANOVA (B) with Bonferroni post-hoc test.

Supplementary Figure S2

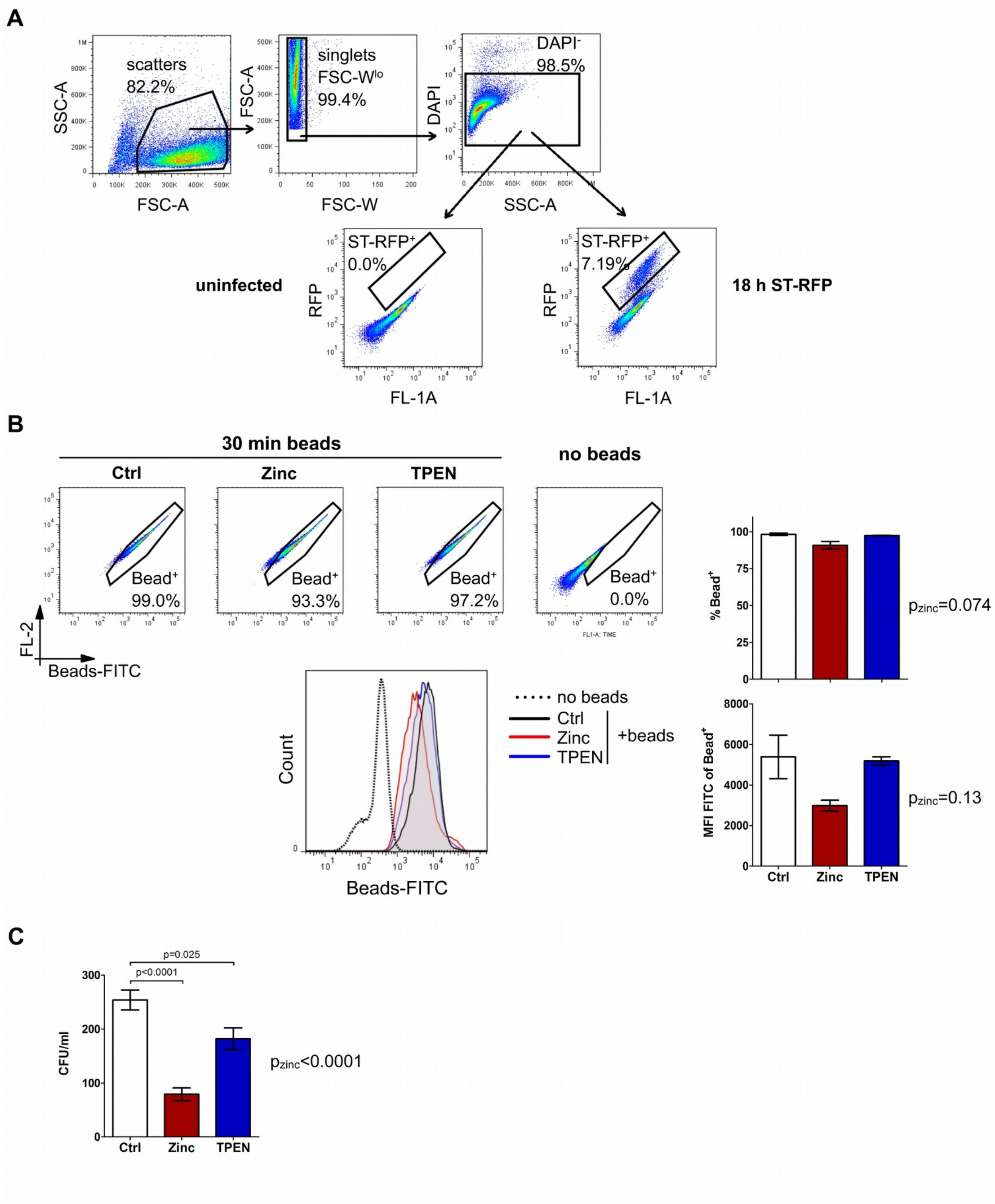

Figure S2. Identification of Salmonella-hosting macrophages by flow cytometry. Phagocytosis capabilities of macrophages upon free zinc modulation. (A) Strategy applied to identify cells hosting viable *Salmonella* by flow cytometry. Analogical strategies were used for detection of GFP-

and RFP-expressing reporter bacteria and for FACS sorting. (B) RAW264.7 cells were were stimulated with vehicle, zinc or TPEN for 4 hours. Subsequently cells were treated with 1:1000 FITC-labeled latex beads (Polysciences) for additional 30 minutes. Uptake of the beads was measured by flow cytometry using a similar strategy to the one presented for reporter bacteria strains. Percent of bead-positive cells and MFI of the bead signal were calculated. Representative data are shown with summary graphs (n=2). (C) RAW264.7 cells were were stimulated with vehicle, zinc or TPEN for 4 hours. Subsequently cells were infected with Salmonella for 30 minutes and lysed. CFU count was determined by plating of whole cell lysates. Statistical significance was calculated with one-way ANOVA with Bonferroni post-hoc test.

Supplementary Figure S3

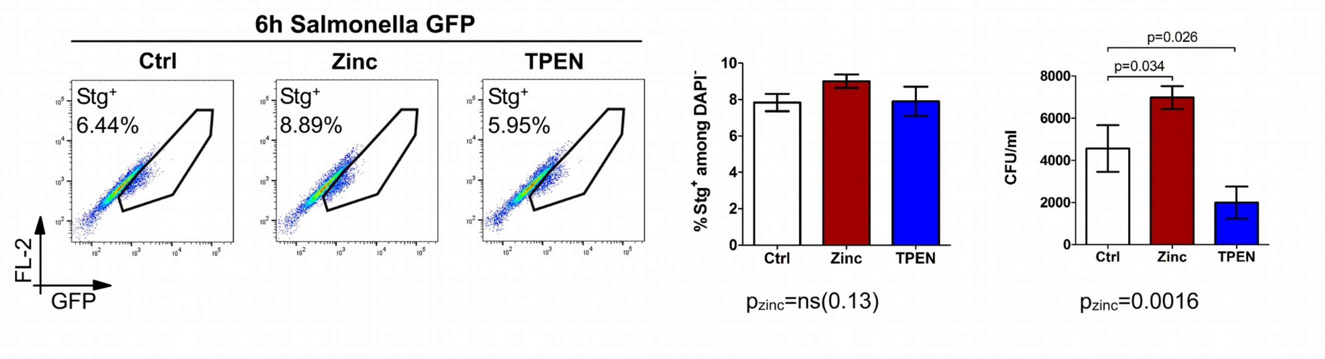

Figure S3. Effects of cellular zinc level modulation on infection rate and anti-microbial defense in bone marrow-derived macrophages (BMDMs). Mouse WT BMDMs (n = 4 mice) were infected with GFP-expressing *Salmonella* Typhimurium (Stg) at MOI=10 and, at the same time, stimulated with 100 µM $ZnCl_2$ or 4 µM TPEN for six hours. Infection rate was measured by flow cytometry among $DAPI^-$ viable macrophages and confirmed by plating of the culture and CFU determination. Representative data are shown. Graphs depict mean with SEM. Graphs depict mean with SEM. Statistical significance was assessed with one-way ANOVA with Bonferroni post hoc tests.

Supplementary Figure S4

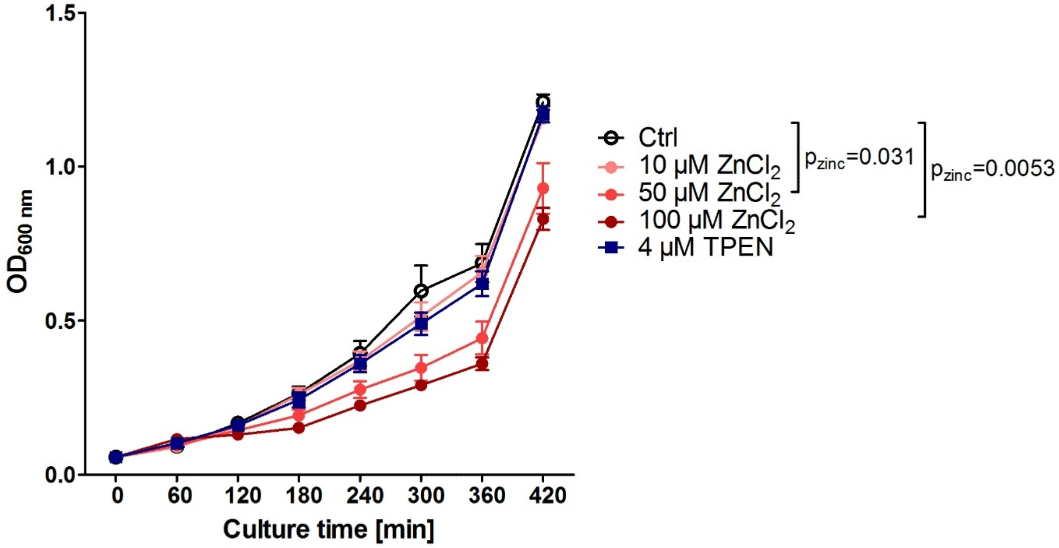

Figure S4. Effects of zinc addition and chelation on bacterial growth in vitro. Bacterial cultures of Wild-Type *Salmonella* Typhimurium were set up as described in Supplementary Methods and supplemented with the indicated concentrations of $ZnCl_2$ and TPEN. Bacterial growth was estimated by spectrophotometric measurements at 600 nm performed every hour. Results of a representative experiment out of two performed are shown. Statistical significance was determined with two-way repeated measure ANOVA; the significances for the main effect of zinc are shown.

Supplementary Figure S5

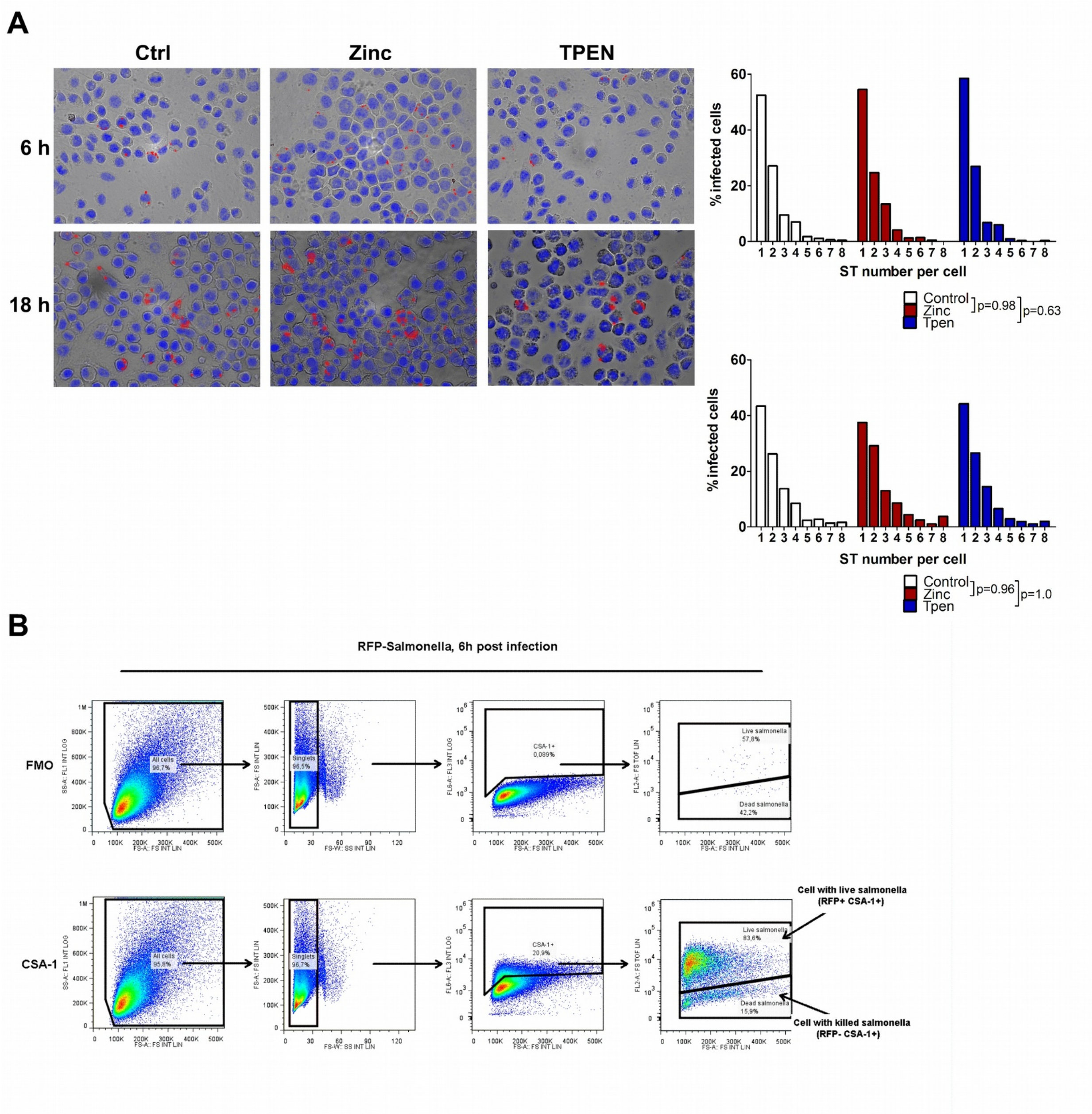

Figure S5. Intracellular proliferation of Salmonella and strategy for identification of cells hosting viable bacteria and cells clearing the pathogen with flow cytometry. (A) RAW264.7 cells were stimulated with vehicle, zinc or TPEN and concomitantly infected with RFP-expressing *S.* Typhimurium for the indicated time points. Number of bacteria per infected cell were determined by manual counting (>500 infected macrophages) of fluorescence microscopy images. Representative

photos are shown. Histograms present distribution of bacteria number per cell. Statistical significance was assessed with Kolmorgov-Smirnov test for distribution differences. (B) RAW264.7 cells were infected with RFP-expressing *S.* Typhimurium, fixed, permeabilized and stained for the intracellular bacteria with the anti-CSA1 antibody recognizing live and recently cleared pathogens. Viable *Salmonella* are defined as $CSA1^{+}$ RFP^{+}, dead bacteria as $CSA1^{+}$ RFP^{-}.

Supplementary Figure S6

A

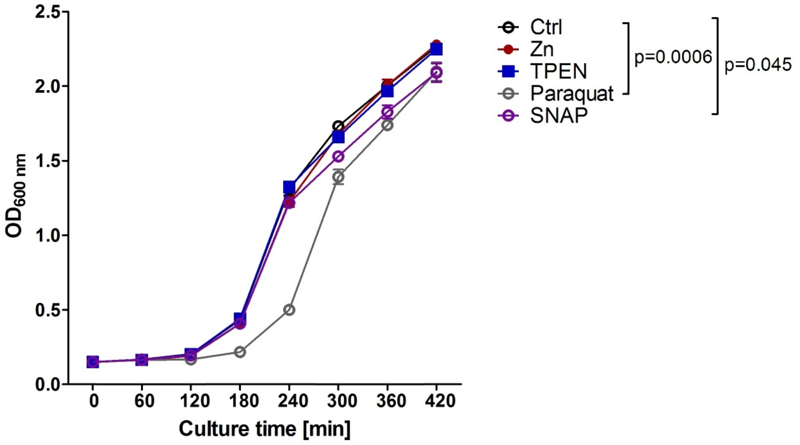

B

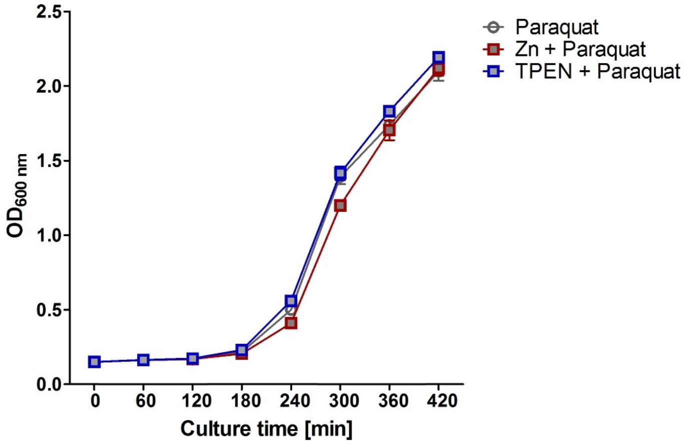

C

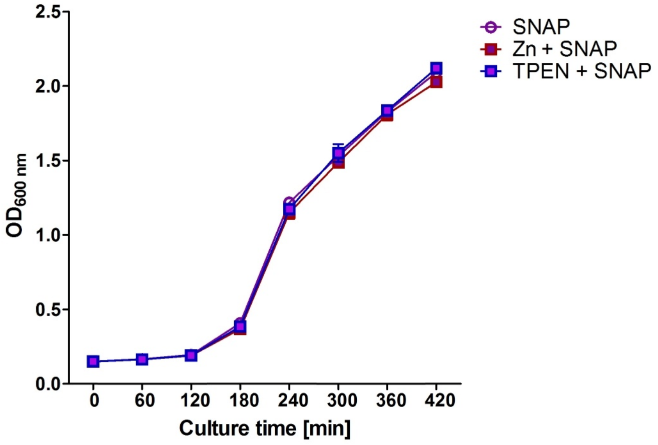

Figure S6. Influence of zinc modulation on resistance of *Salmonella* Typhimurium to oxidative and nitrosative stress. Bacterial cultures were supplemented with $ZnCl_2$ (100 µM), TPEN (4 µM), the ROS generator paraquat (500 µM) and the NO donor S-Nitroso-N-acetyl-DL-penicillamine (SNAP, 500 µM) and combinations thereof. Bacterial growth was estimated by spectrophotometric measurements at 600 nm performed every hour. Results of a representative experiment out of two performed are shown. Statistical significance was determined with two-way repeated measure ANOVA; the significances for the main effect of zinc are shown. (A) Comparison of growth curves in controls and cultures stimulated with zinc, TPEN, paraquat and SNAP. (B) Bacterial growth under stimulation with paraquat and zinc modulation. (C) Bacterial growth under stimulation with SNAP and zinc modulation.

Supplementary Figure S7

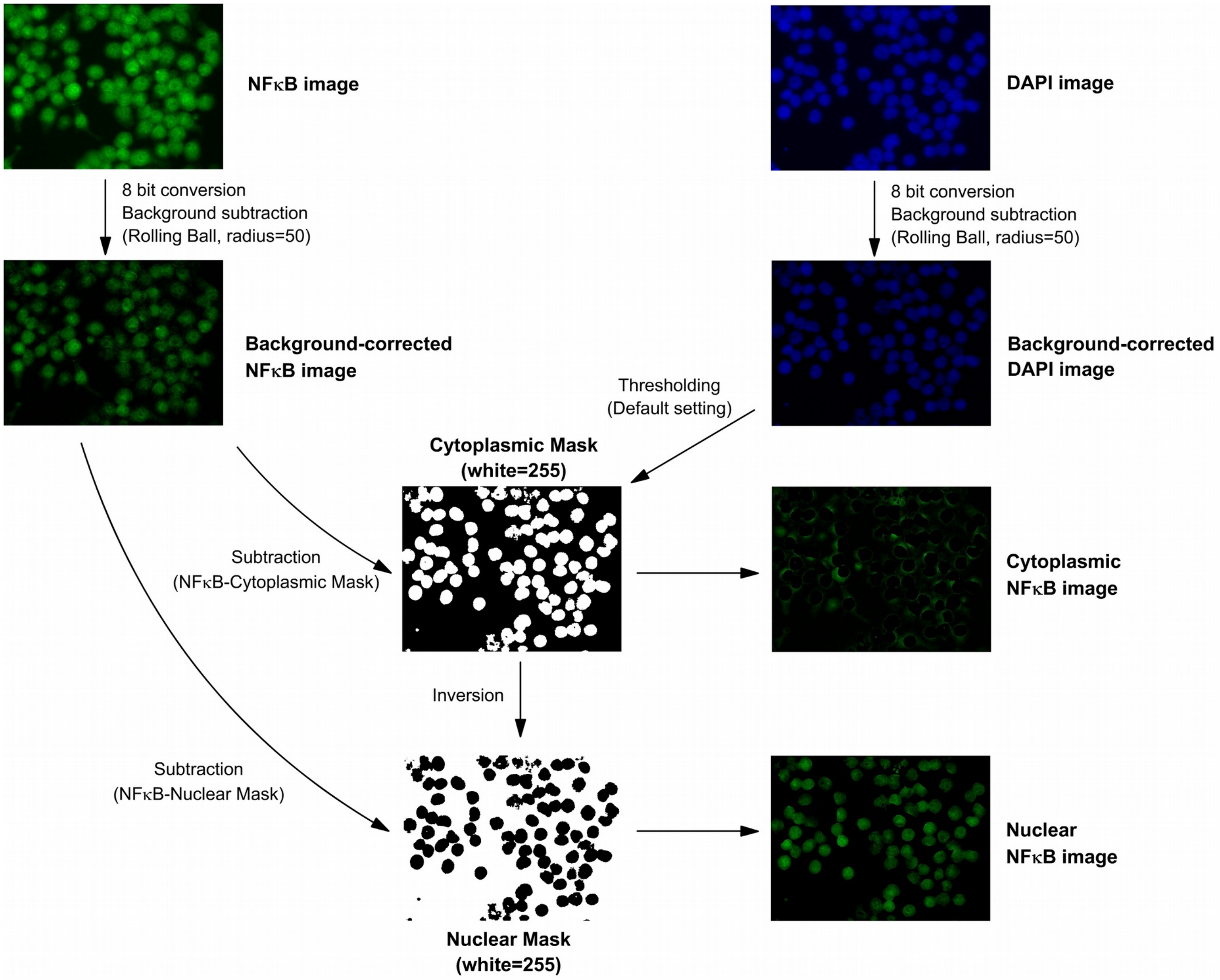

3Figure S7. Analysis of fluorescence microscopy data to determine nuclear and cytoplasmatic NF-κB signal intensities. RAW264.7 cells were infected with *S.* Typhimurium, fixed, permeabilized and stained for total NF-κB and DNA (DAPI). The graphics depicts following steps in image correction and extracting nuclear and cytoplasmic signal images. Mean signal intensity of the nuclear and cytoplasmic signal images was calculated and presented in Figure 5. All image operations were performed with ImageJ software.

Supplementary Figure S8

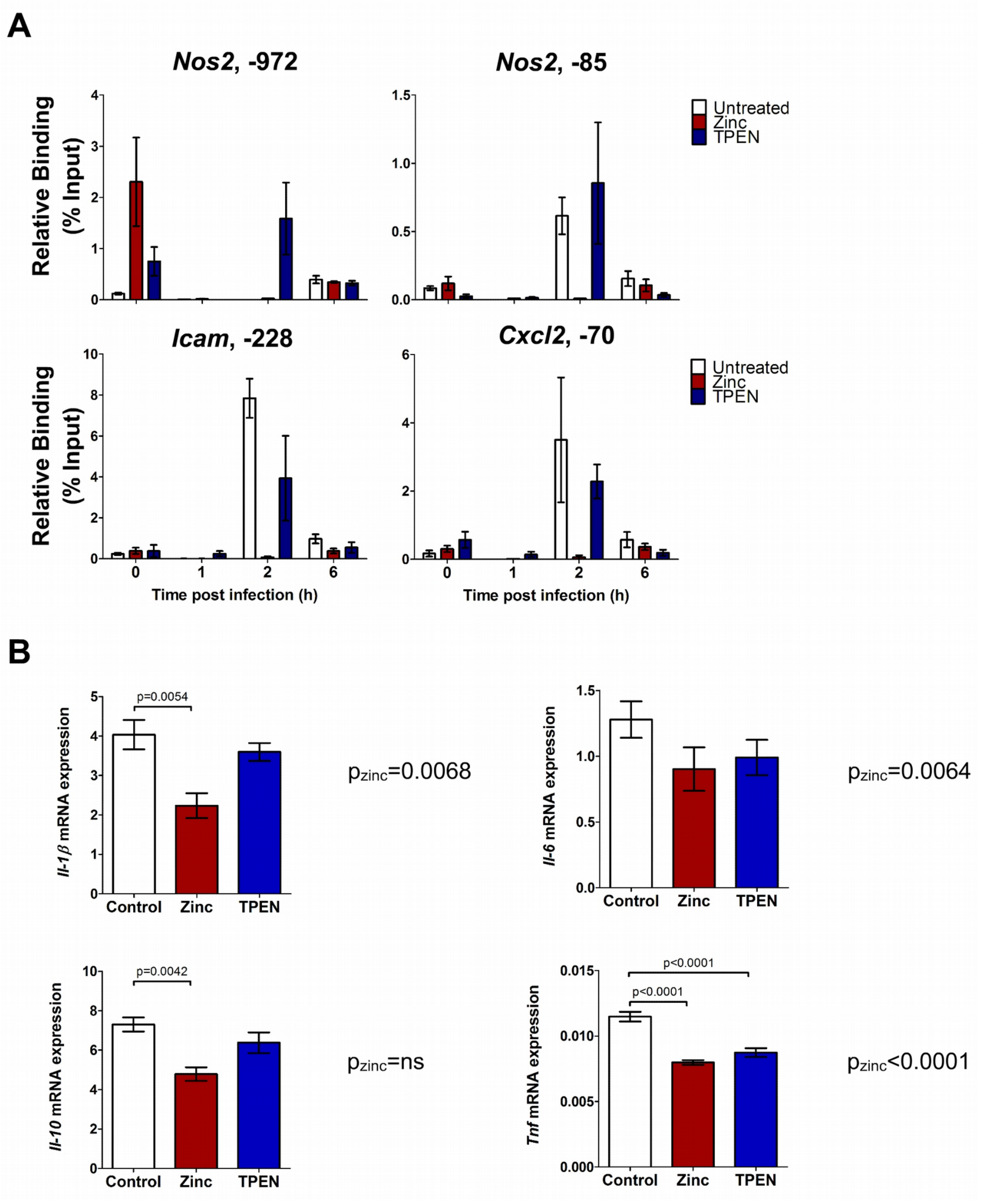

Figure S8. NF-κB transcriptional activity and expression of NF-κB target genes. RAW264.7 cells were stimulated with vehicle, zinc or TPEN and infected with *S.* Typhimurium for the indicated time points. (A) Chromatin immunoprecipitation with anti-pSer536 NF-κB antibodies was performed and occupancy of the described promoter sites in *Nos2*, *Icam* and *Cxcl2* genes was measured by Real-Time

PCR. Data obtained in a representative experiment (n=2) with technical duplicates are shown. (B) Transcript levels for *Il-1b*, *Il-6*, *Il-10* and *Tnf* genes were determined with qRT PCR 6 h after *Salmonella* infection (n=4). Statistical significance was calculated with two-way ANOVA with Bonferroni post-hoc test.

Supplementary Figure S9

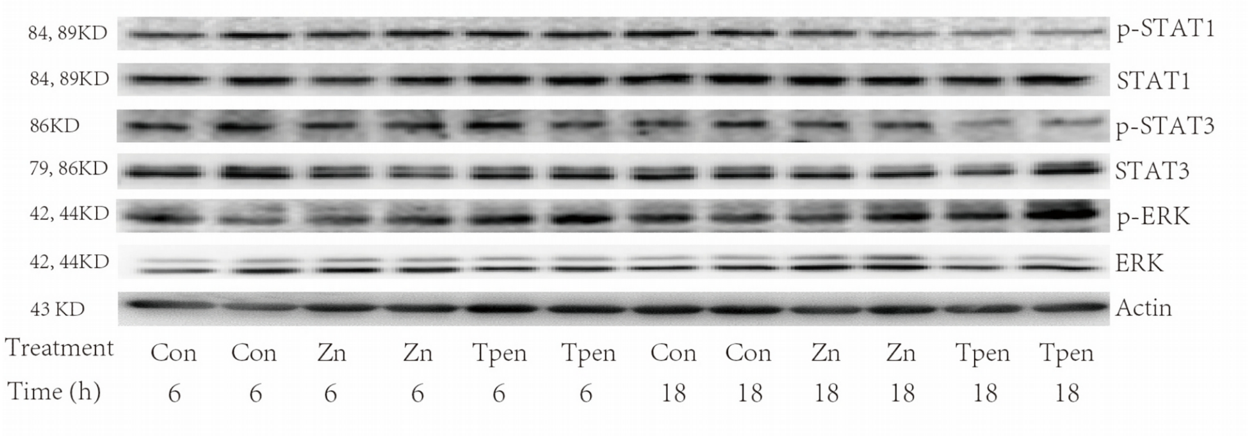

Figure S9. Activation of STAT and ERK signaling pathways in Salmonella-infected macrophages upon zinc modulation. RAW264.7 cells were infected with *S.* Typhimurium for the indicated time points. Activity of STAT1, STAT3 and ERK signaling pathways was investigated with Western Blotting technique. Results of a representative experiment out of two performed is shown.

Supplementary Figure S10

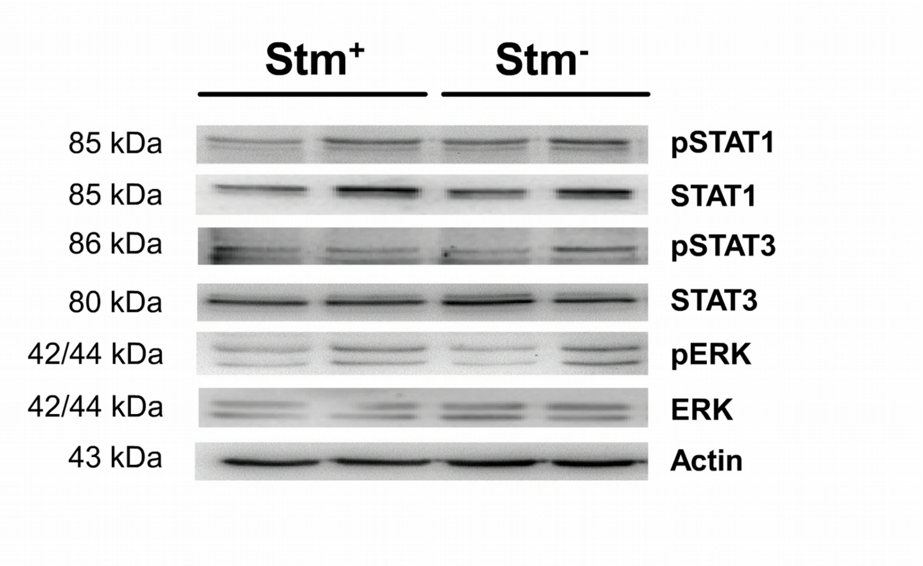

Figure S10. Activation of STAT and ERK signaling pathways in *Salmonella*-hosting and *Salmonella*-clearing/non-infected macrophages. RAW264.7 cells were infected with GFP-expressing *S.* Typhimurium for 6 h. Cells containing viable *Salmonella* (GFP^{+}) and macrophages without living bacteria (GFP^{-}) were FACS sorted. Activity of STAT1, STAT3 and ERK signaling pathways was investigated with Western Blotting technique. Results of a representative experiment out of two performed is shown.

Supplementary Figure S11

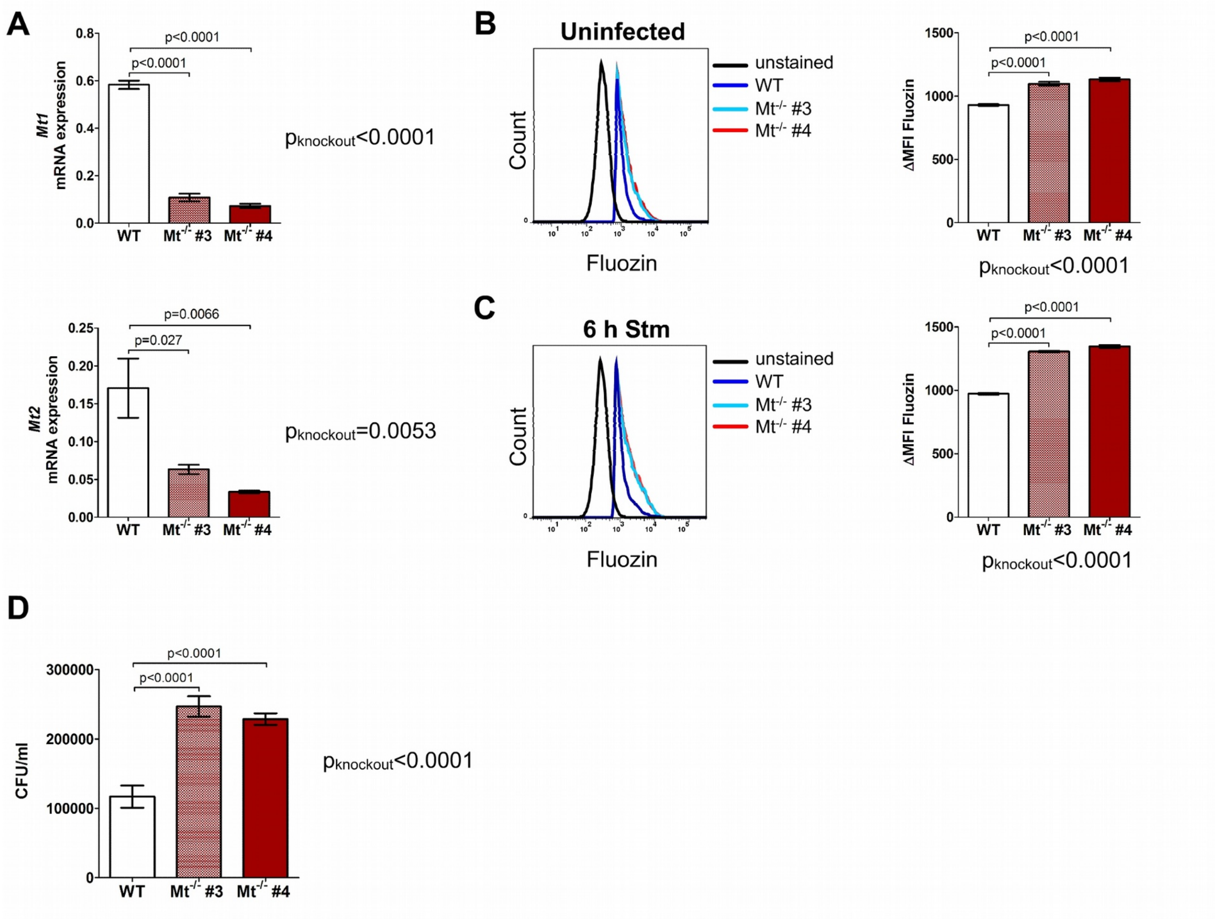

Figure S11. Free zinc accumulation in Mt1/2$^{-/-}$ macrophages. Infection rate in WT and Mt1/2$^{-/-}$ macrophages measured by plating. Double knockout Mt1/2$^{-/-}$ cells and parental WT RAW264.7 cells were infected with GFP-expressing *S.* Typhimurium for the indicated time points. (A) Knockout efficiency in uninfected Mt$^{-/-}$ #3 and Mt$^{-/-}$ #4 macrophages was determined with qRT PCR for *Mt1* and *Mt2* transcripts (n=4). (B, C) Free zinc levels were measured by Fluozin staining and flow cytometry in uninfected cells (B) and macrophages infected with *Salmonella* for 6 hours (C). The plot depicts ΔMFIs (n=3). (D) WT, Mt$^{-/-}$ #3 and Mt$^{-/-}$ #4 RAW264.7 cells were infected with *S.* Typhimurium or 12 hours. Subsequently cells were lysed and lysates plated to determine CFU counts (n=6). Statistical significance was calculated with one-way ANOVA with Bonferroni post-hoc test.
